# Comparison of the vitality tests used in the dental clinical practice and histological analysis of the dental pulp

**DOI:** 10.17305/bjbms.2021.6841

**Published:** 2022-02-10

**Authors:** Ana Tenyi, Lidija Nemeth, Aljaž Golež, Ksenija Cankar, Aleksandra Milutinović

**Affiliations:** 1 Department of Dental Diseases and Normal Dental Morphology, Medical Faculty University of Ljubljana, Ljubljana, Slovenia; 2 Institute of Physiology, Medical Faculty University of Ljubljana, Ljubljana, Slovenia; 3 Institute of Histology and Embryology, Medical Faculty University of Ljubljana, Ljubljana, Slovenia; 4 International Center for Cardiovascular Diseases MC Medicor Slovenia, Ljubljana, Slovenia.

**Keywords:** Pulse oximetry, electrical sensibility test, histology, volume density of blood vessels and myelinated nerve fibers

## Abstract

In dentistry, indirect diagnostic methods such as electrical sensibility testing and pulse oximetry are used to assess the status of the pulp. Our study aimed to determine the correlation between hemoglobin oxygen saturation and vascular volume density (Vvasc). We also wanted to examine an electrical sensibility test and the volume density of myelinated nerve fibers (Vnerv). Twenty-six intact permanent premolars were included in the study. For histological analysis, the pulp tissue was stained with hematoxylin-eosin and immunohistochemically for von Willebrand factor and S100 to detect blood vessels and myelinated nerve fibers, respectively. The stereological analysis was used to determine the Vvasc and Vnerv. Statistical analysis was done using the Pearson correlation test and Welch’s ANOVA test. Histological analysis showed that the pulp tissue was strongly vascularized and innervated. A significant positive correlation was found between Vvasc and hemoglobin oxygen saturation levels (p = 0.030). A significant negative correlation was found between Vnerv and the lowest electrical voltage that patient felt (p = 0.033). According to the maturity of the dental apex, teeth were divided into a group with open (n = 6, OA group) and closed apex (n = 20, CA group). We found that pulps in the CA group had higher Vnerv than the OA group (p = 0.037). In contrast, there were no significant differences in Vvasc of the pulp tissue (p = 0.059), oxygen saturation (p = 0.907), or electrical voltage (p = 0.113) between both groups. We can conclude that the measurement of pulse oximetry and electrical sensibility test reflects the morphology of healthy pulp tissue independently of the maturity of the dental apex.

## INTRODUCTION

An accurate assessment of dental pulp vitality in dental clinical practice carries pivotal importance since this determines whether the dentist might opt for a more conservative dental treatment, or more radical ones, such as endodontic therapy, need to be performed. For this estimation, standard sensibility tests to thermal (usually cold) or electric stimuli are adopted for clinical use [1]. Unfortunately, such tests provide only indirect and subjective information, which depends first on the individual’s perceived response to stimuli and second on the clinician’s interpretation of the patient’s sensory perception. In addition, results of the sensibility test are often unconvincing or inconclusive with other clinical findings, which make the setting of the diagnosis even more difficult [2].

A considerable disadvantage of sensibility testing methods using cold or electric stimuli is that assessing an authentic pulp vitality cannot be measured since these methods do not evaluate pulpal vascular circulation, only its neural response [3]. In comparison to its innervation, the vascular supply of dental pulp is a more accurate indicator of proper pulp vitality [4].

The electric pulp tester is a device commonly used for applying a known electric current to the inspected tooth, to stimulate the closest myelinated A-delta fibers in the dental pulp [3]. Positive response to such current only indicates the viable neural transmission and the presence of vital nerve fibers, but does not prove the health of the tissue or assess any potential damage of the vascular supply of the pulp [5].

On the other hand, with the use of a non-invasive pulse oximetry monitoring, hemoglobin oxygen saturation in the dental pulp can be successfully recorded [6]. Pulse oximetry, based on principles of spectrophotometry and optical plethysmography, serves as an objective test that is not painful nor requires any response from the patient [7]. Similarly to pulse oximetry, the method of laser Doppler flowmetry [5] and other innovative techniques are being described and further developed for clinical use of pulpal vitality assessment [8,9].

Histologically, the dental pulp tissue is richly innervated and strongly vascularized [5]. The pulp tissue has the highest density of unmyelinated C fibers that innervate the pulped body of the pulp. Myelinated A-fibers innervate the periphery of the pulp tissue. About 90% of them are thin myelinated A-delta fibers, and the least represented are myelinated A-beta fibers [10]. Compared to C fibers, A-delta fibers have a lower electrical threshold and respond more quickly to stimuli [11].

The electric pulp tester is a device to apply an electric current to the tooth surface. It stimulates the intact thin myelinated A-delta fibers that innervate the periphery of the pulp [3]. The response to stimulation is the most significant in the area of the tooth surface above the pulp horn because there is the highest concentration of axons [5,12]. A positive response to electrical stimulation only indicates the presence of vital nerve fibers, but does not demonstrate tissue health or assess possible vascular pulp supply damage [5].

Dental pulp tissue is known to be highly vascularized [13]. Blood volume represents a high percentage of the wet weight of pulp tissue [14]. One method for estimating dental pulp vascularity is pulse oximetry [5].

Pulse oximetry is a non-invasive method that, based on spectrophotometry and optical plethysmography, allows monitoring of hemoglobin oxygen saturation in dental pulp [6]. It is an objective method, not painful, and does not require any response from the patient [7].

In medicine, histopathological examinations are used to confirm the presence and type of a particular disease. Histological diagnostic methods cannot be used in routine dental practice, as the tooth would be destroyed when a pulp sample is taken for examination.

Consequently, dentists are left to rely on the results of diagnostic devices, and although the sensibility of these tests is high, with false-positive or false-negative results, the consequences can severely affect the teeth prognosis [15]. A tooth that has been falsely diagnosed as non-vital by an electric pulp tester could be subjected to unnecessary pulp removal and root canal treatment, while a tooth that is falsely diagnosed as vital may remain untreated, resulting in necrosis of supporting tissues and their resorption [1]. In addition, with recent advances in vital pulp therapy and the tendency for the preservation of pulp vitality [16,17] or its regeneration [18,19], it is of the outmost importance for clinical diagnosis to correlate to the actual pulpal status [20].

The aim of our study was to determine a possible correlation between the pulse oximetry values as a test of pulp vitality and the density of blood vessels. We also wanted to examine the standard electric sensibility test and the density of myelinated nerves at the top of the pulp.

## MATERIALS AND METHODS

### Patients and tests of pulp vitality and sensibility

Twenty-six permanent upper and lower premolars of seven patients, aged from 12 to 20 years, were included in the study. All teeth were healthy without any visible signs of defects (caries, erosions, cracks, wear, etc.). The teeth were scheduled for extraction due to orthodontic indication. Before extractions, clinical examinations with measurements of hemoglobin oxygen saturation using pulse oximetry (SpectrO2, Smiths Medical, OH, USA) and electrosensibility tests (CICADA Tech. Dev. Co., Foshan, Guangdong, China) were performed by a single investigator, as described previously [7,21,22]. To avoid any potential disruption in pulpal blood circulation due to electrical stimulation, both tests were carried out at different appointments.

**FIGURE 1:**
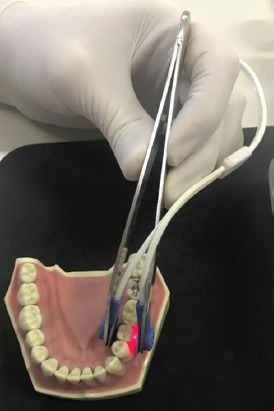
“Y” sensor on each branch of dental forceps placed on the dental crown.

Hemoglobin oxygen saturation levels were recorded using the customized pulse oximetry monitor, connected to its “Y” sensor, and placed parallel onto the each of the branch of dental forceps, as shown in Figure 1. This way it was possible to achieve close contact between detector and the tooth surface. One branch of the forceps, holding the light-emitting sensor, was placed on the labial surface of premolar and the other on the oral surface of the same tooth. Each tooth that was evaluated was first cleaned and dried with a cotton ball and later isolated with cotton rolls. Since vascularity of the gingival tissue is much greater than that of the pulp, pulse oximetry signals originating in the gingiva easily overwhelm the pulpal effects. To maximally attenuate the signals received by measuring the oxygen saturation of the gingival vascular supply (or totally eliminate the effect of acquisition of false positive results), only the crown of the tooth was carefully placed between the forceps and gingival tissue was properly isolated. For the importance of attaining undisturbed signal, special attention was drawn to maintaining parallel alignment of both diodes and avoiding any patient movements. Such close contact was then secured for at least 45 s, until the value of saturation (% SpO_2_) on the monitor was stable and remained unchanged. To obtain the results of pulse oximetry as accurate as possible, a finger sensor of another pulse oximetry device was attached to the patient’s finger for the entire time of the measuring.

Electrical pulp testing was carried out at a separate appointment using an electric pulp tester (CICADA Tech. Dev. Co., Foshan, Guangdong, China) with a separate electrode for the attachment to the patient’s lower lip. Toothpaste was applied as the conducting medium and the probe was placed on an intact tooth occlusal surface. The first (lowest) positive patients’ response at the lowest voltage applied was recorded as the voltage value (mV), shown on the monitor of the tester.

### Tissue samples and staining

**FIGURE 2:**
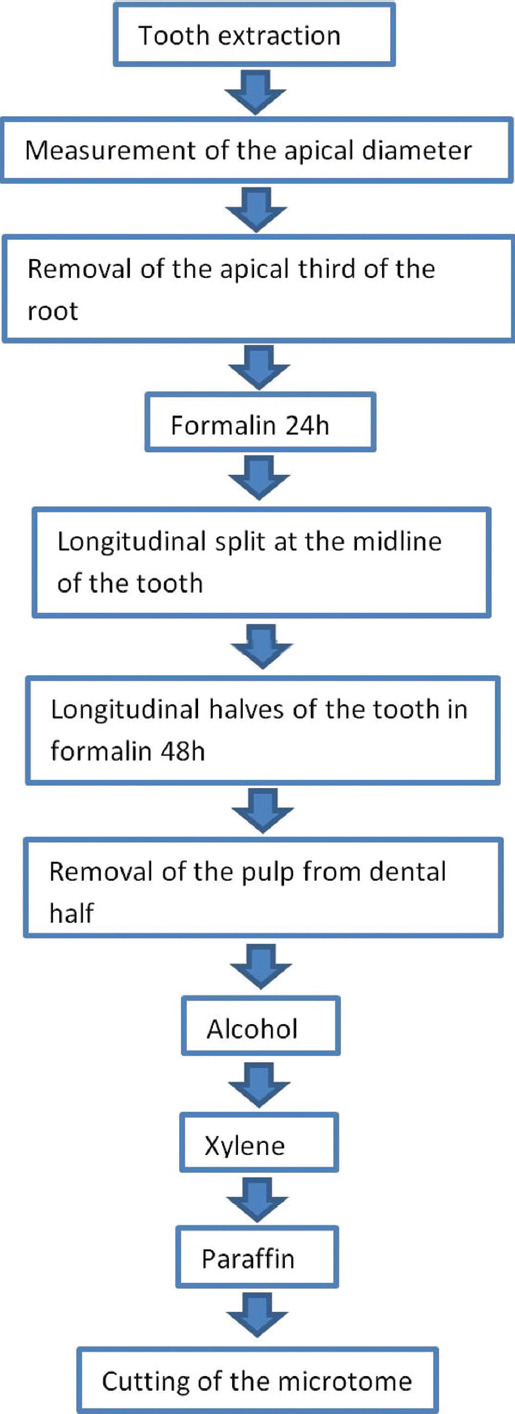
The preparation process of the extracted teeth.

The preparation process of the extracted teeth is shown in Figure 2. Immediately after extraction, apices of all teeth were examined to assess the size of their apical diameter. The diameter of the apical foramen was measured using an endodontic hand file instrument with a known standardized dimension.

Following this, one-third of the apical part of the root was cut off for better penetration of the fixating solution into the pulp tissue and fixed in formalin for 24 hours [23]. After 24 hours, the entire tooth’s vertical (longitudinal) split was done. The halves of the tooth with pulp were re-immersed in formalin for another 48 hours. Then, the pulps were gently removed from the dental half, dehydrated in alcohol, immersed in xylene, embedded in paraffin, and cut into 4.5 mm thick longitudinal step serial sections. The step between the two sections was 20 mm thick. Sections were stored at room temperature and stained with HE. Blood vessels were shown by immunohistochemical labeling of endothelial cells with anti-von Willebrand factor (vWf) or factor 8-related antigen (Dako Denmark, 1:800) [24].

Myelinated A-delta fibers were shown immunohistochemically by staining myelin sheaths with anti-S-100 (Dako Denmark, 1:1000). It was reported that much more prominent immunoreactivity of S100 appeared in the cytoplasm of myelin-forming Schwann cells than Schwann cells surrounding unmyelinated axons [25]. The immunohistochemistry was done following the manufacturer’s instructions as described previously [26,27].

### Image analysis and evaluation of the volume density of blood vessels (Vvasc.) and myelinated nerve fibers (Vnerv.)

Image analysis was performed under a light microscope (Nikon Eclipse E 400), using a camera (Nikon digital sight DS-M5) and NIS elements version 3 – documentation computer program. The measurements were performed on three sagittal slices of the central part of the dental pulp at the objective magnification of ×40 for blood vessels in the crown part of the pulp tissue. The objective magnification of ×60 for the myelinated nerve fibers was used for measurement in the sub-odontoblast zones on the occlusal side of the pulp.

The volume density of blood vessels lumen and myelinated nerve fibers was stereological analyzed using the Weibel’s test system described previously [28].

### Teeth with open and closed apices

According to the diameter of the dental apex, teeth were divided into two groups: Teeth with open (n = 6, OA group) and closed apex (n = 20, CA group). The apices with <0.015 mm diameter were defined as closed (n = 20) and more than 0.04 mm as open (n = 6). None of the teeth enrolled in the study had apices’ diameters between 0.015 mm and 0.04.

### Ethical statement

The Slovenian national medical ethics committee approved the study under protocol number 0120 – 415/2020/6. All invited participants received proper information before procedures and signed the informed consent form.

### Statistical analysis

Analyses were performed using Microsoft Excel 2010 and Statistical Package for the Social Sciences SPSS 20.

The sample size was determined using the power of the study at 0.8 and *p*-value significance at <0.01. The result for appropriate sample size was n = 19. To compensate the potential dropout, the sample size was set at 26.

The relationship between the volume density of blood vessels and oxygen saturation and the volume density of nerve fibers and electrical voltage was tested by Pearson’s coefficient of correlation (*p* < 0.05).

The average values of the measured parameters (volume density blood vessels and myelinated nerve fibers, oxygen saturation, and electrical voltage) for the CA (n = 20) and OA group (n = 6) were calculated and expressed as the average value ± SD. ANOVA and the Welch test (*p* < 0.05) evaluated the statistical significance of the differences between both groups to compare unequally sized samples.

## RESULTS

### Patients and extracted teeth

Twenty-six, normal healthy upper and lower permanent premolars of seven patients, were included in the study. Six teeth of two patients (both girls aged 12 years) had an open apex and 20 teeth of five patients (three girls aged from 15 to 20 and two boys aged 13 and 15) had closed apex. The characteristics of teeth are listed in Table 1.

**TABLE 1:**
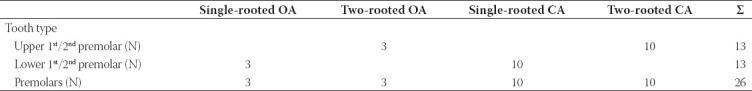
Distribution of specimen included in the study, regarding the tooth type, number of teeth, and apex width

### Tissue samples

**FIGURE 3:**
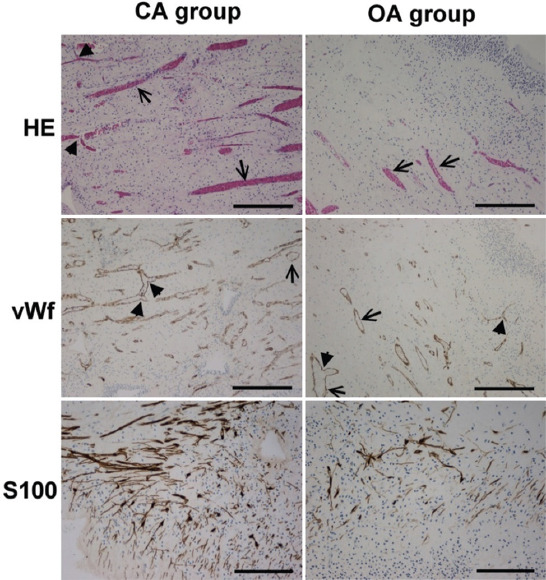
Crown part of dental pulp with closed (CA group) and open apex (OA group) stained with HE (objective magnification ×4, bar=300μm), anti-vWf (objective magnification ×4, bar=300μm), and anti-S100 (sub-odontoblast zone, objective magnification ×20, bar=150μm). Note that the pulp tissue is insignificantly more vascularized (HE, vWf) and significantly more innervated (S100) in the CA group than in the OA group. ↑: Vessels with the thin wall; ▲: Branching of the vessels in the right angels.

Histological examination of the pulps stained with HE and immunohistochemically for vWf and S100 (Figure 3) showed the loose connective tissue in the central part of the pulp. The peripheral part consisted of the odontoblast and sub-odontoblast zones, composed of a cell-free (Weil zone) and a cell-rich layer (Hohl zone). The odontoblast zone was often torn from the dentin during pulp isolation. The pulp tissue had many blood vessels with a thin wall. The main arterioles, venues, and nerves run parallel to the long axis of the tooth. They were located in the central part of the root canal. Blood vessels branched many times at the right angles. Arteries that run from these branches often had the caliber greater than the vessel they originated. Transversal branches between vessels were also seen. In coronary pulp, the axons and blood vessels extensively branched. The density of capillary bad and nerves plexus increased toward the upper part of the dental pulp in the crown.

### Correlation between vascular volume density and hemoglobin oxygen saturation

**FIGURE 4:**
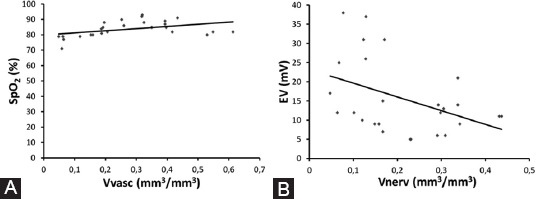
(A) Linear correlation between the vascular volume density of the pulp tissue (Vvasc) and levels of hemoglobin oxygen saturation (SpO_2_) (R=0.426, *p*=0.030, Pearson correlation); (B) linear correlation between the volume density of nerve fibers (Vnerv) and the lowest electrical voltage (EV) felt by the patient (R=−0.420, *p*=0.033, Pearson correlation).

The average value of vascular volume density was 0.276 mm^3^/mm^3^ ± 0.159 and oxygen saturation 83.731% ± 5.166. The Pearson’s test revealed a significant positive correlation between vascular volume density of the pulp tissue and levels of hemoglobin oxygen saturation measured through pulse oximetry (r = 0.426, *p* = 0.030) (Figure 4A).

### Correlation between myelinated nerve fibers volume density and voltage

The average value of volume density of nerve fibers was 0.218 mm^3^/mm^3^ ± 0.115 and the lowest electrical voltage that patient felt, measured with an electric pulp tester was 15.615 mV ± 9.798. We found a significant negative correlation between the volume density of nerve fibers and voltage (r = −0.420, *p* = 0.033) (Figure 4B).

### Comparison between CA and CO group in volume density of blood vessels and myelinated nerve fibers, results of pulse oximetry, and electrical sensibility test

**FIGURE 5:**
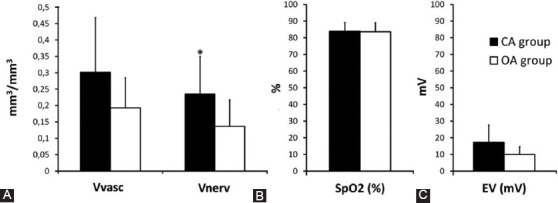
The differences between the CA and OA groups in (A) vascular (Vvasc) and nerve fibers (Vnerv) volume density of the pulp tissue; (B) oxygen saturation (SpO_2_) and (C) the lowest electrical voltage (EV) felt by the patient. (*Significantly different; ANOVA, Welch test [*p*<0.05]).

According to the maturity of the dental apex, teeth were divided into CA (n = 20) and OA (n = 6). The results of vascular and nerve volume density, oxygen saturation, and electro-voltage of dental pulps of the CA and OA groups are represented in Figure 5.

We found that pulps in the CA group had a higher density of nerve fibers in the area beneath the odontoblast zone (0.234 mm^3^/mm^3^ ± 0.116) than the group of teeth with open apices (0.137 mm^3^/mm^3^ ± 0.080; *P* = 0.037) (Figures 3 and 5A).

In contrast, there was no significant difference in vascular volume density of the pulp tissue between both groups (CA group: 0.301 mm^3^/mm^3^ ± 0.167 and OA group: 0.193 mm^3^/mm^3^ ± 0.092; *p* = 0.059) (Figures 3 and 5A).

In addition, there was also no difference in hemoglobin oxygen saturation (CA group: 83.80 % ± 5.238 and OA group: 83.50 % ± 5.394; p = 0.907) (Figure 5B) or in minimal electric voltage that patients felt (CA group: 17.30 mV ± 10.378, and OA group: 10.00 mV ± 6.648; *p* = 0.113), between both groups of teeth (Figure 5C).

## DISCUSSION

In this study, healthy teeth, extracted due to orthodontic indication, were investigated using clinical tests to assess whether the vitality and sensibility of dental pulp are actually reflected in its morphology.

We found a positive correlation between the volume density of blood vessels and the pulp tissue’s oxygen saturation levels independently on the apex’s maturity. The dental pulp is known to be very vascular tissue [13]. Blood volume was reported to represent about 3% of the wet weight of pulp tissue in rats, similar to breast tumor tissue [14]. In young cats, it was reported that 14% of dental pulp volume is occupied by vessels, and the average density of the capillary network was found to be 1402 mm^2^ [29,30]. The vascular bed in our study was also dense, with very thin walls to their diameters which were also observed by the previous studies [13,14].

One of the methods for *in vivo* showing the vascularity of dental pulp is pulse oximetry [5]. It is a clinical method for monitoring the oxygen saturation of the hemoglobin. Oxygen saturation of blood in tissue is a reflection of the dynamic balance between oxygen supply and consumption in capillaries, arterioles, and venues. It is generally accepted that oxygen saturation is associated with blood perfusion [31].

It is known that pulp vascularity [32] and oxygen saturation [5,22,33,34] are higher in the younger people’s teeth and that they decrease with age. In a study involving 120 healthy premolars, pulse oximetry showed decreased oxygen saturation in the dental pulp after the age of 35, but after the age of 40, the reduction in oxygen saturation was significant [34]. In our study, participants were aged from 12 to 20 years, and the average oxygen saturation of premolars was much lower (83.73%) in the groups of patients aged 20-24 years, compared to the previously mentioned study (89.71%). Our results of pulp oxygenation are similar to measurements of oxygenation of 40-year-old patients (80%) from reports mentioned in the previous study. However, their results agree with the results of a histological study of the dental pulp of 120 patients, where a decrease in the vascular density was shown, regarding with increase of years of life. The significant drop was especially visible between 30 and 40 years of age [32].

In the other study, the dental pulp’s oxygen saturation by pulse oximetry was measured on incisors with open and closed apex. The results showed a higher average value of oxygen saturation in teeth with open apex (approximately 85%) in comparison to teeth with closed ones (approximately 83%). They found a negative correlation between the stage of tooth root development and saturation with oxygen. In our study, the pulps were divided into CA and OA groups according to the maturity of the apex. Both groups were very similar in oxygen saturation values; CA group 83.80% and OA group 83.50%. Our values were in a very similar range to the previous study on incisors [33] but lower than the study on premolars [34]. However, we found no significant difference in vascular density of the pulp tissue between the CA and OA groups. Moreover, in the pulps of the CA group, an insignificantly higher vascular volume density was noted than in the OA group. Significantly higher blood flow in teeth with newly closed apex than open apex was shown in a study where blood flow was measured using transmitted-light plethysmography at different stages of tooth root development [35]. Further studies should be done, as the blood vessels of the dental pulp have not yet been fully investigated.

It is known that in addition to rich vascularity, the dental pulp tissue is also highly innervated [10]. Our study observed that the axons in the crown part of the pulp were extensively branched, but the axons in the radicular part gave off only a few branches that have also been reported by other authors [10,36]. Mechanical, chemical, thermal, and electrical stimulation of exposed dentin or dental pulp elicit just sensations of pain [10]. The lowest electrical potential through hard dental tissues causes excitation in delta myelinated A-delta nerve fibers. The result is a sensory perception that can be measured by an electrical sensibility test [5,37].

Our study found a significant negative correlation between the volume density of myelinated nerve fibers and the lowest electrical voltage that the patient felt. We also found a significantly higher volume density of nerve fibers in the CA group than in the OA group. Our results agree with the previously described findings that the pulp of developing teeth has a less dense network of nerve fibers, especially myelinated fibers, than in a fully formed young tooth. It was also reported that the young developing teeth are less sensitive to pain than adult teeth [38,39], but we did not find significant differences in the electric test of sensibility between both groups. In our experiment, teeth sensibility to electric current in the OA group was even insignificantly higher. In the OA group, we only had six teeth out of two patients, resulting in a significant impact of individual differences in pain perception [2], and we attributed deviations in our results from those reported in the literature to the subjectivity of our two subjects.

According to our knowledge, just a few studies analyzed pulp histology and compared it with clinical tests. In the 1960s and 1970s, the association between pulp histological status (normal, inflamed, and necrotic) and the result of the electrical sensibility test was investigated. However, no significant correlation was found [37,40-43], except the association between necrotic pulp and the absence of sensation in the electrical test [37,40].

We need to be careful in interpreting our results, as not many patients were included in the study, especially in the OA group. The latter is the major limitation of this study. However, the results of our study are important as they show the results of clinical tests and histological analysis of the same teeth in humans.

## CONCLUSION

We found that healthy teeth with a closed apex had a higher volume density of nerve fibers in the upper part of the dental pulp than intact teeth with an open apex, but there were no differences in the perception of the lowest electrical voltage felt by the patient as there are large individual differences in sensitivity. However, we found no significant differences in dental pulp vascularity and oxygen saturation of dental pulp tissue between teeth with closed and open apex, confirming the findings that pulse oximetry is a more objective method than electrical sensibility test.

Regardless of the openness of the apex, we found a positive correlation between the volume density of blood vessels and the degree of saturation of pulp tissue with oxygen. We also found a significant negative correlation between nerve fiber volume density and the lowest electrical voltage felt by the patient. We can conclude that the measurement of pulse oximetry and electrical sensibility test reflects the morphology of healthy pulp tissue independently of the maturity of the dental apex.

## References

[1] Sigurdsson A (2003). Pulpal diagnosis. Endod Top.

[2] Mejàre IA, Axelsson S, Davidson T, Frisk F, Hakeberg M, Kvist T (2012). Diagnosis of the condition of the dental pulp:A systematic review. Int Endod J.

[3] Alghaithy RA, Qualtrough AJ (2017). Pulp sensibility and vitality tests for diagnosing pulpal health in permanent teeth:A critical review. Int Endod J.

[4] Abd-Elmeguid A, Yu DC (2009). Dental pulp neurophysiology:Part 2. Current diagnostic tests to assess pulp vitality. J Can Dent Assoc.

[5] Gopikrishna V, Pradeep G, Venkateshbabu N (2009). Assessment of pulp vitality:A review. Int J Paediatr Dent.

[6] García AA, Forner L, Sanz J, Llena C, Lozano FJ, Guerrero-Gironés J (2021). Pulse oximetry as a diagnostic tool to determine pulp vitality:A systematic review. Appl Sci.

[7] Anusha B, Madhusudhana K, Chinni SK, Paramesh Y (2017). Assessment of pulp oxygen saturation levels by pulse oximetry for pulpal diseases-a diagnostic study. J Clin Diagn Res.

[8] Lin CY, Chen F, Hariri A, Chen CJ, Wilder-Smith P, Takesh T (2018). Photoacoustic imaging for noninvasive periodontal probing depth measurements. J Dent Res.

[9] Mozaffarzadeh M, Moore C, Golmoghani EB, Mantri Y, Hariri A, Jorns A (2021). Motion-compensated noninvasive periodontal health monitoring using handheld and motor-based photoacoustic-ultrasound imaging systems. Biomed Opt Express.

[10] Hossain MZ, Bakri MM, Yahya F, Ando H, Unno S, Kitagawa J (2019). The role of transient receptor potential (TRP) channels in the transduction of dental pain. Int J Mol Sci.

[11] Olgart L (1974). Excitation of intradental sensory units by pharmacological agents. Acta Physiol Scand.

[12] Byers MR, Dong WK (1983). Autoradiographic location of sensory nerve endings in dentin of monkey teeth. Anat Rec.

[13] Iijima T, Zhang JQ (2002). Three-dimensional wall structure and the innervation of dental pulp blood vessels. Microsc Res Tech.

[14] Berggreen E, Bletsa A, Heyeraas K (2010). Circulation in normal and inflamed dental pulp. Endod Top.

[15] Halappa M (2015). Pulp vitality tests-an overview on pulp vitality and sensitivity. Indian J Oral Sci.

[16] Taha NA, About I, Sedgley CM, Messer HH (2020). Conservative management of mature permanent teeth with carious pulp exposure. J Endod.

[17] Ricucci D, Loghin S, Siqueira JF (2014). Correlation between clinical and histologic pulp diagnoses. J Endod.

[18] Kim SG, Malek M, Sigurdsson A, Lin LM, Kahler B (2018). Regenerative endodontics:A comprehensive review. Int Endod J.

[19] Khan SZ, Mirza S, Karim S, Inoue T, Bin-Shuwaish MS, Al Deeb L (2020). Immunohistochemical study of dental pulp cells with 3D collagen Type I gel in demineralized dentin tubules *in vivo*. Bosn J Basic Med Sci.

[20] Mickel AK, Lindquist KA, Chogle S, Jones JJ, Curd F (2006). Electric pulp tester conductance through various interface media. J Endod.

[21] Bhaskar SN, Rappaport HM (1973). Dental vitality tests and pulp status. J Am Dent Assoc.

[22] Gopikrishna V, Tinagupta K, Kandaswamy D (2007). Evaluation of efficacy of a new custom-made pulse oximeter dental probe in comparison with the electrical and thermal tests for assessing pulp vitality. J Endod.

[23] Daud S, Nambiar P, Hossain MZ, Saub R, Ab Murat N, Mohamed A (2016). Removal of the apical one-third of the root improves the fixation process of the dental pulp in teeth. J Histotech.

[24] Jacoby BH, Davis WL, Craig KR, Wagner G, Farmer GR, Harrison JW (1991). An ultrastructural and immunohistochemical study of human dental pulp:Identification of Weibel-Palade bodies and von willebrand factor in pulp endothelial cells. J Endod.

[25] Mata M, Alessi D, Fink DJ (1990). S100 is preferentially distributed in myelin-forming Schwann cells. J Neurocytol.

[26] Hasanović A, Mornjaković Z, Pikula B, Dilberović F (2006). Morphologic findings of the ischemic myocardium. Bosn J Basic Med Sci.

[27] Radović S, Dorić M, Zujo H, Hukić A, Kuskunović S, Babić M (2012). Interdigitating dendritic cell sarcoma of the liver and lung:A case report with morphohological and immunohistochemical features of tumor. Bosn J Basic Med Sci.

[28] Milutinovic A, Zorc-Pleskovic R (2012). Glycogen accumulation in cardiomyocytes and cardiotoxic effects after 3NPA treatment. Bosn J Basic Med Sci.

[29] Matthews B, Andrew D (1995). Microvascular architecture and exchange in teeth. Microcirculation.

[30] Vongsavan N, Matthews B (1992). The vascularity of dental pulp in cats. J Dent Res.

[31] Wang G, Jia S, Liu M, Song X, Li H, Chang X (2020). Impact of local thermal stimulation on the correlation between oxygen saturation and speed-resolved blood perfusion. Sci Rep.

[32] Baker A, Karpagaselvi K, Kumaraswamy J, Ranjini MR, Gowher J (2019). Role of dental pulp in age estimation:A quantitative and morphometric study. J Forensic Dent Sci.

[33] Bargrizan M, Ashari MA, Ahmadi M, Ramezani J (2016). The use of pulse oximetry in evaluation of pulp vitality in immature permanent teeth. Dent Traumatol.

[34] Estrela C, Serpa GC, Alencar AHG, Bruno KF, Barletta FB, Felippe WT (2017). Oxygen saturation in the dental pulp of maxillary premolars in different age groups-Part 1. Braz Dent J.

[35] Ganbold K, Kakino S, Ikeda H, Miyashin M (2017). Human pulpal blood flow in different root formation stages measured with transmitted-light plethysmography. Arch Oral Biol.

[36] Daud S, Nambiar P, Hossain MZ, Rahman MR, Bakri MM (2016). Changes in cell density and morphology of selected cells of the ageing human dental pulp. Gerodontology.

[37] Jafarzadeh H, Abbott PV (2010). Review of pulp sensibility tests. Part II:Electric pulp tests and test cavities. Int Endod J.

[38] Fried K, Gibbs J (2014). Dental Pulp Innervation, NYU Scholars.

[39] Yu C, Abbott PV (2007). An overview of the dental pulp:Its functions and responses to injury. Aust Dent J.

[40] Reynolds RL (1966). The determination of pulp vitality by means of thermal and electrical stimuli. Oral Surg Oral Med Oral Pathol.

[41] Mumford JM, Bowsher D (1976). Pain and protopathic sensibility. A review with particular reference to the teeth. Pain.

[42] Matthews B, Searle BN, Adams D, Linden R (1974). Thresholds of vital and non-vital teeth to stimulation with electric pulp testers. Br Dent J.

[43] Cooley RL, Robison SF (1980). Variables associated with electric pulp testing. Oral Surg Oral Med Oral Pathol.

